# Direct and correlated responses to selection for autumn lambing in sheep

**DOI:** 10.1186/s12711-020-00577-z

**Published:** 2020-10-02

**Authors:** Masood Asadi-Fozi, Heather L. Bradford, David R. Notter

**Affiliations:** 1 Department of Animal and Poultry Sciences, Virginia Tech, Blacksburg, VA 24061 USA; 2 Department of Animal Science, Faculty of Agriculture, Shahid Bahonar University of Kerman, 76169-133 Kerman, Iran

## Abstract

**Background:**

Seasonal reproduction limits productivity, flexibility, and profitability in commercial sheep production. Hormonal and (or) photoperiodic manipulation can be used to control estrous cycles in sheep and reduce limitations that are imposed by the seasonal anestrous but are often impractical or incompatible with the extensive management systems preferred for ruminant livestock. Thus, the current study investigated the use of selection to improve realized fertility (i.e., the proportion of ewes that lambed) following an out-of-season spring joining period (May and June) in a crossbred sheep population.

**Results:**

Over 17 years, estimated breeding values (EBV) for fertility in selected (S) ewes increased by 0.175 (0.01 per year). The mean EBV for fertility of S ewes was greater than that of control ewes by year 10 (P = 0.02), and the fertility of adult (≥ 3 years old) ewes reached 0.88 ± 0.05 by year 17. Lambing began approximately 140 days after the introduction of rams, and 64% of the S ewes that lambed did so in the first 17 days of the potential lambing season, which indicated that most of the S ewes were cycling at the time of ram introduction and were not induced to cycle by the introduction of breeding males (i.e., the so-called “ram effect”). Animals in the S line had modest increases in body weight and scrotal circumference. A modest negative trend in the additive maternal effect on birth weight was observed but was reversed by additional selection on EBV for maternal birth weight. The heritability of litter size in autumn lambing was low (0.04) and could potentially limit the response to selection for this trait.

**Conclusions:**

Selection improved realized ewe fertility in out-of-season mating, with absolute increases of approximately 1% per year in the percentage of joined ewes that lambed in the autumn. Genetic antagonisms with other performance traits were generally small. A modest antagonism with maternal breeding values for birth weight was observed but it could be accommodated by selection on EBV for maternal birth weight. Our results support results of previous studies that indicate that these selected ewes had one of the shortest seasonal anestrous periods reported for temperate sheep breeds and that spring-lambing lactating ewes from the selection line were capable of relatively rapid rebreeding in the spring.

## Background

Seasonal changes in reproductive activity are commonly observed in sheep and goats exposed to the circannual fluctuations in day length that occur at temperate latitudes [1]. A distinct anestrous period is present in essentially all temperate sheep breeds, but with considerable variation in timing and duration of the seasonal anestrous [2]. Breeds that were developed under tropical and subtropical conditions often do not have a well-defined seasonal anestrous but, if moved to higher latitudes, may express a seasonal pattern of reproduction in response to more extreme fluctuations in day length [3]. Seasonal breeding places constraints on commercial sheep producers. The gestation length of the ewe is approximately 145 d, theoretically allowing ewes to lamb every 6 to 8 months, but the seasonal anestrous generally results in a single annual spring lambing. Estrous behavior in the ewe can be modified by controlling photoperiod or administration of gonadotropins and (or) melatonin [4], but these interventions are, in many cases, not feasible with the extensive management that is preferred for ruminant livestock.

Attempts to develop lines of sheep with improved fertility in autumn lambing [5–8] generally did not yield definitive results because of limitations in designs, lack of appropriate control populations, or early termination of the study before experimental objectives were achieved. Thus, the objective of the current study was to use selection to attempt to improve the frequency of autumn (October and November) lambing in a crossbred sheep population managed in an annual autumn-lambing system.

## Methods

### Location and populations

**Fig. 1:**
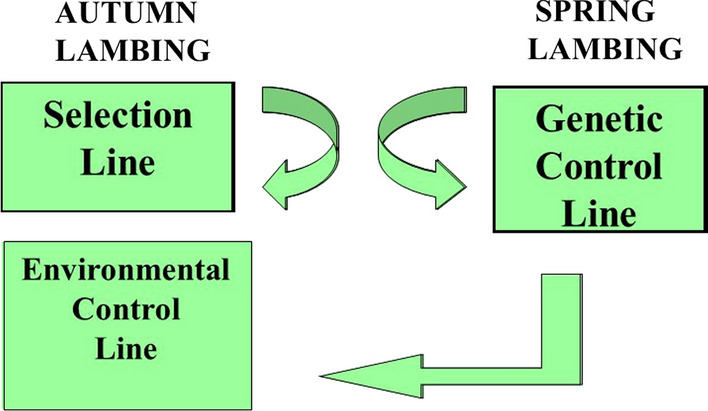
Experimental design to evaluate improvement of autumn lambing by selection [11]. Arrows indicate the flow of replacement ewe lambs in the three lines. Replacement ewes and rams in the S (selection) and GC (genetic control) lines were produced within the lines. Unselected replacement ewes for the EC (environmental control) line were produced in the GC line and transferred to the EC line for evaluation

This study was conducted in Blacksburg, VA at 37° N and 80° W. The base population was a composite line with 50% Dorset, 25% Rambouillet, and 25% Finnsheep breeding [9]. In autumn of 1988, ewes obtained by one to three generations of inter se mating between parents of this composite line were randomly divided within age and sire into a spring-bred selection line (S; n = 125), a contemporary spring-bred environmental control line (EC; n = 55), and an autumn-bred genetic control line (GC; n = 45) (Fig. 1). Two control lines were required to allow control animals to be both randomly selected and contemporary to animals in line S. Progeny of EC ewes could not be randomly selected because they were born to ewes that conceived in the spring. Thus, the GC ewes were mated in autumn and used to produce randomly selected EC replacement ewes, which were moved to the spring-mated EC line. Replacement ewes for the GC and EC lines were chosen at random within GC sires. Thus, the same sires were represented in the EC and GC lines, and full-sib ewe lambs born in the GC line were normally represented in both EC and GC lines. A necessary limitation of this design was that EC ewes, born in spring (March) and joined for the first time in May at 14 months of age, were an average of 7 months older than ewes in line S at each joining.

### Mating scheme

**Fig. 2:**
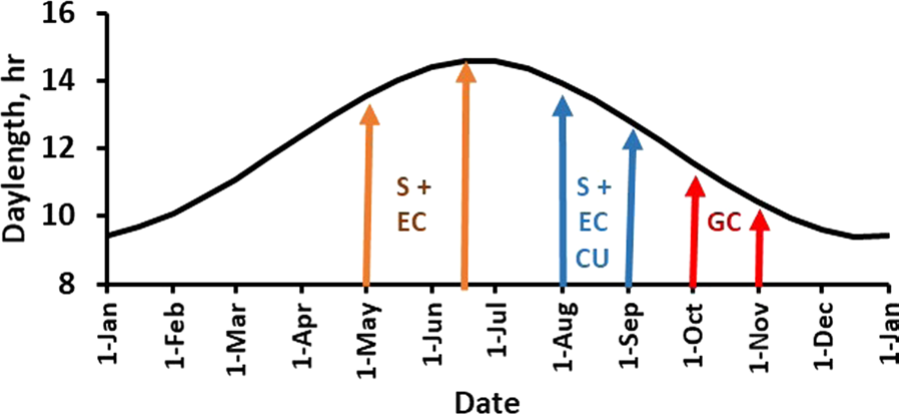
Annual changes in day length at Blacksburg, Virginia, USA (37.2° N, 80.4° W). Arrows show the beginning and end of the joining period for selection (S), environmental control (EC), and genetic control (CG; red arrows) lines. Tan and blue arrows indicate the primary and clean-up (CU) joinings, respectively for S and EC ewes

The S and EC ewes were joined with rams during 6 weeks from May 1, and GC ewes were joined with rams during 4 weeks from October 1. The selection objective was to improve fertility during the annual spring joining period. However, to avoid keeping potentially large numbers of barren ewes, S and EC ewes were also joined with groups of Suffolk rams during 30 d from August 1. Lambs from this joining period were born in January and weaned in early March. We assumed that S and EC ewes that had lambed in January would not be less fertile than ewes that had lambed in October at the subsequent May joining period. Figure 2 shows the periods of joining for the three lines and annual changes in day length at the experimental location. One third of the ewes in lines S and EC were replaced each year in order to maintain comparable distributions of ewe age for both lines. Half of the rams in line S were replaced each year, with no more than two sons from the same sire. Rams in line S were generally used for one or two years, but occasionally retained for a third year. By contrast, GC ewes and rams were replaced only when necessary because of death or health issues in order to maximize the generation interval and effective population size. These replacement rates were maintained rigorously throughout the experiment.

The experiment was divided into three phases: 1, 2 and 3. In Phase 1 (1989 through 1993) [10, 11], S and EC ewes were exposed to vasectomized rams during two weeks before mating began on May 1. The S ewes were joined with one of 10 S rams, and EC ewes were joined with one of five GC rams in single-sire breeding pens. Selection of replacement ewes and rams in line S was based on the mean fertility of their dams during the spring joining period (1 for ewes that lambed in autumn and 0 for ewes that did not lamb; hereafter referred to only as “fertility”), with emphasis on keeping the progeny from the small number of ewes that lambed successfully in autumn at 12 months of age.

Phase 2 (1994 through 1998) included modifications based on the results from Phase 1 [11]. Based on the generally acceptable fertility achieved in Phase 1, ewes were no longer exposed to vasectomized rams before joining. In addition, S and EC ewes were all joined with S rams in 10 single-sire breeding pens. This modification allowed a more direct comparison of fertility for S and EC ewes but also allowed S males to potentially affect the mating behavior of EC ewes. Selection decisions in Phase 2 were based on estimated breeding values (EBV) for fertility using parameters from Phase 1 [10].

In Phase 3 (1999 through 2005), selection continued in the S line, but the GC line was terminated, and no new replacements were added to the EC line. Selection procedures in Phase 3 were the same as those in Phase 2. However, subsamples of S and remaining EC ewes were used in several semi-intensive studies [12–16], in which ewes were not given the opportunity to lamb in one or more years, thereby reducing selection intensity in the S line. However, fertility data were collected for all available S ewes in 2004 and 2005.

### Animal management and data collection

Ewes in all lines were maintained throughout the year on grass-legume pastures that comprise primarily tall fescue, orchardgrass, and white clover. Grass-legume hay was fed as needed in January through March. Ewes received alfalfa hay and a supplement of grain in late gestation and during lactation. Lambs were weighed and docked within 24 h of birth. Male lambs remained intact. Lactating ewes and their lambs were maintained in an elevated feeding facility with an expanded-metal floor. Lambs had access to creep feed after approximately 3 weeks of age and were weighed and weaned at approximately 60 d of age. Lambs remained in the same facility, were fed a high-concentrate diet ad libitum for 60 d after weaning, and were weighed at approximately 90 and 120 d of age. Scrotal circumference was measured on male lambs at approximately 60, 90, and 120 d of age but was not recorded in 1991. Replacement rams and ewes were identified by 150 d of age, removed from the feeding barn, and managed on grass-legume pastures with a supplement of grain as needed to achieve target minimum breeding weights of 30 kg for females and 50 kg for males at approximately 210 d of age. All lambs of S ewes that attained the target breeding weight and were free of obvious structural defects were retained and joined with rams. The number of replacement S ewes was reduced to approximately 42 (i.e., one third of the S ewe flock) before the second joining period by removing yearling ewes that did not lamb and had the lowest average dam fertility (Phase 1) or EBV for fertility (Phases 2 and 3). Housing, management, and data collection for lactating GC ewes and their lambs were the same as for autumn-born lambs. Most GC ewe lambs were retained to meet replacement needs for the GC and EC flocks.

The intensity of lamb performance recording was relaxed in some years during Phase 3. Lambs were weighed only at 60 and 120 d in 2001, and 60 and 90 d in 2003. In 2002, 15% of the available ram lambs were identified after weaning as possible replacements based on EBV for fertility; 120-d weights were recorded for these ram lambs and all ewe lambs, but the remaining ram lambs were not retained. In 2004, 82% of the male lambs and 30% of the female lambs were removed from the study after recording weaning and 90-d BW. In 2005, 23% of the lambs of both sexes were removed after recording weaning and 90-d BW. Measurement of SC was discontinued after 2000.

Autumn-born ram lambs selected for use in the spring at 7 to 8 months of age were subjected to an examination for breeding soundness a few days before joining. In small pens, the ram lambs were exposed to adult ewes that had been brought into estrus. Ram lambs that did not serve any ewes on the first day of testing were held overnight in pens adjacent to those containing estrus ewes and tested again on the following day. Twenty five to 35% of the candidate ram lambs did not serve estrus ewes and were discarded.

### Statistical methods

#### Data

For this study, data on fertility and litter size came only from spring joining periods of S and EC ewes. Records of reproductive performance for spring-lambing GC ewes were used only to characterize their fertility during the autumn joining period. Likewise, birth weight records came only from autumn-born S and EC lambs; this decision was based on differences in direct and maternal heritabilities for birth weight between lambs born in autumn and spring [17]. Postnatal BW were recorded under the same intensive management and feeding in autumn and spring, and BW records from S, EC, and GC lambs were included in analyses of postnatal BW. However, since photoperiod could potentially affect the rate of testis growth, records of SC were limited to autumn-born lambs. Ewe and lamb records obtained from matings to Suffolk sires in August were not used in any of the analyses. Means and numbers of observations for each variable in the final dataset are in Table 1.

**Table 1:** Descriptive statistics and data structure for ewe reproduction and lamb performance

Item	Ewe reproduction		Lamb performance						
	Fertility^a^	Litter size^b^	Birth weight	60-day BW	90-d BW	120-day BW	60-day SC	90-day SC	120-day SC
Mean	0.49	1.75	3.6	19.7	28.8	37.2	13.6	20.0	25.7
Standard deviation	0.50	0.60	0.9	5.0	6.4	8.2	2.3	3.9	3.4
Maximum	1	1	6.4	35.5	48.2	60.6	20.7	30.8	32.5
Minimum	0	3	1.8	12.7	25.7	31.1	8.2	10.5	15.5
Number of records^c^	2956	1443	2286	2493 (1858)	2082 (1536)	1312 (1312)	472	469	422
Number of ewes or dams^d^	1048	591	588	541	484	467	247	250	230
Number of ewe or lamb sires^d^	141	133	102	101	99	94	75	75	72
Average. number of records per ewe or dam^d^	2.82	2.44	4.13	3.43	3.17	2.81	1.91	1.88	1.83

^a^Realized fertility during the spring joining period = 1 for ewes that lambed in autumn and 0 for ewes that did not lamb

^b^Number of lambs born per ewe lambing

^c^Number of records for lamb BW is for both autumn- and spring-born lambs or, in parentheses, only autumn-born lambs; all other variables used only lambs born in autumn

^d^Number of ewes, number of ewe sires, and records per ewe for ewe reproduction; number of dams, number of dam sires, and records per dam for lamb performance

*BW* body weight (kg), *SC* scrotal circumference (cm)

#### Models and parameter estimation

Genetic parameters and EBV were estimated using Bayesian methods, multivariate animal models, and the BLUPF90 software [18]. The additive relationship matrix ($\mathbf{A}$) included 4921 animals from the three lines and extended back to the founders of the base population [9]. Animals in the S, GC, and EC lines were derived from a common base population, and line effects were not included in the statistical models. Analyses used 1,000,000 Gibbs samples, discarded 20,000 burn-in samples, and retained every 500th sample. Burn-in was determined based on visual diagnostics. Thinning was set to keep most of the autocorrelations lower than 0.5 and effective sample sizes larger than 30. Fertility was analyzed with both linear and threshold models.

The model for ewe fertility and litter size was:

$${\mathrm{Y}}_{\mathrm{ijkl}}={\mathrm{T}}_{\mathrm{i}}+{\mathrm{C}}_{\mathrm{j}}+{\upbeta }_{1}{\mathrm{F}}_{\mathrm{k}}+{\upbeta }_{2}{\mathrm{F}}_{\mathrm{k}}^{2}+{\mathrm{A}}_{\mathrm{k}}+{\mathrm{PE}}_{\mathrm{k}}+{\mathrm{P}}_{\mathrm{il}}+{\mathrm{e}}_{\mathrm{ijkl}},$$

where ${\mathrm{Y}}_{\mathrm{ijkl}}$ is the fertility or litter size, ${\mathrm{T}}_{\mathrm{i}}$ and ${\mathrm{C}}_{\mathrm{j}}$ are fixed effects of lambing year and ewe age class, respectively; ${\upbeta }_{1}$ and ${\upbeta }_{2}$ are continuous linear and quadratic effects of inbreeding ($\mathrm{F}$) of the $\mathrm{k}$^th^ animal; ${\mathrm{A}}_{\mathrm{k}}$ and ${\mathrm{PE}}_{\mathrm{k}}$ are random additive animal and permanent environmental effects, respectively, for the $\mathrm{k}$^th^ animal; ${\mathrm{P}}_{\mathrm{il}}$ is the random effect of the $\mathrm{l}$^th^ service sire in the $\mathrm{i}$^th^ lambing year; and ${\mathrm{e}}_{\mathrm{ijkl}}$ is a random residual. Random effects of $\mathrm{A}$, $\mathrm{PE}$, $\mathrm{P}$, and $\mathrm{e}$ were assumed to be uncorrelated. Ewe age classes distinguished between yearling, 2-year-old and adult (more than 3 years old) ewes. The yearling ewe age class was further subdivided to discriminate between ewes that were allowed to lamb for the first time at 12 (S) or 19 (EC) months of age. The S and EC ewes were placed in the same ewe age classes after the first lambing opportunity. Heritabilities and repeatabilities for these traits were estimated from univariate models as the ratios of additive genetic and additive genetic plus ewe permanent environmental variances, respectively, to phenotypic variance.

Lamb birth weight and BW and SC at 60, 90, and 120 days were analyzed as separate traits. Before analysis, birth weights were adjusted for dam age and type of birth and postnatal BW and SC were adjusted for effects of dam age and type of birth and rearing using multiplicative adjustment factors derived from the data and following procedures from [19]. The model was:

$${\mathrm{W}}_{\mathrm{ijkl}}={\mathrm{TS}}_{\mathrm{i}}+{\mathrm{X}}_{\mathrm{j}}+{\upbeta }_{3}{\mathrm{D}}_{\mathrm{k}}+{\upbeta }_{4}{\mathrm{F}}_{\mathrm{k}}+{\upbeta }_{5}{\mathrm{F}}_{\mathrm{l}}+{\mathrm{A}}_{\mathrm{k}}+{\mathrm{M}}_{\mathrm{l}}+{\mathrm{MPE}}_{\mathrm{l}}+{\mathrm{L}}_{\mathrm{il}}+{\mathrm{e}}_{\mathrm{ijkl}},$$

where ${\mathrm{W}}_{\mathrm{ijkl}}$ is the observed BW or SC; ${\mathrm{TS}}_{\mathrm{i}}$ are the fixed effects of lambing year for birth weight and SC or lambing year and season (autumn or spring) for postnatal BW; ${\mathrm{X}}_{\mathrm{j}}$ are the fixed effects of lamb sex (omitted for SC); ${\upbeta }_{3}$ is a continuous linear effect of lamb age ($\mathrm{D}$; omitted for birth weight); ${\upbeta }_{4}$ and ${\upbeta }_{5}$ are continuous linear effects of inbreeding ($\mathrm{F}$) of lamb and dam, respectively; ${\mathrm{A}}_{\mathrm{k}}$ and ${\mathrm{M}}_{\mathrm{l}}$ are random direct and maternal additive effects, respectively, for the $\mathrm{k}$^th^ animal and the $\mathrm{l}$^th^ dam; ${\mathrm{MPE}}_{\mathrm{l}}$ is the permanent environmental effect of the $\mathrm{l}$^th^ dam; ${\mathrm{L}}_{\mathrm{il}}$ is a random litter effect; and ${\mathrm{e}}_{\mathrm{ijkl}}$ is the random residual. For all models, additive direct-maternal covariances could not be estimated and were set to zero, and non-genetic random effects (i.e., $\mathrm{MPE}$ and $\mathrm{L}$) were assumed to be uncorrelated with each other and with other random effects. In addition, models for SC that contained both additive maternal and dam permanent environmental effects failed to converge and did not yield reasonable estimates for these variance components. Based on the deviance information criterion (DIC) [20], the maternal permanent environmental effect was removed from the final models for SC. Estimates of additive genetic (${\sigma }_{\mathrm{A}}^{2}$), additive maternal (${\sigma }_{\mathrm{M}}^{2}$), maternal permanent environmental (${\sigma }_{\mathrm{MPE}}^{2}$), litter (${\sigma }_{\mathrm{L}}^{2}$), and residual (${\sigma }_{\mathrm{e}}^{2}$) variances were expressed as proportions of phenotypic variances (${{\sigma }_{\mathrm{P}}^{2}=\sigma }_{\mathrm{A}}^{2}+{\sigma }_{\mathrm{M}}^{2}+{\sigma }_{\mathrm{MPE}}^{2}+{\sigma }_{\mathrm{L}}^{2}+{\sigma }_{\mathrm{e}}^{2}).$

#### Genetic trends

Estimated breeding values for fertility were derived for univariate linear and threshold models. Mean EBV for fertility were calculated by year and line and were based on the ewes that were joined in each line and year or, for line S, the average EBV of each joined pair [11]. This approach was preferable to estimates of genetic change being based on average EBV of lambs born in each line because ewes that did not conceive in spring did not produce lambs and EC lambs were sired by S rams in Phase 2. Four multi-trait analyses were used to estimate genetic parameters, covariances with fertility (modeled as a threshold trait), and EBV of joined ewes for (1) ewe litter size, (2) lamb birth weight, (3) lamb BW at 60, 90, and 120 d, and (4) SC in male lambs at 60, 90, and 120 d. Ewes that did not lamb did not have records for litter size or lamb performance and residual correlations between these traits and fertility were set to zero. Likewise, when fertility was correlated with traits with an additive maternal effect (i.e., lamb BW and SC), covariances between additive effects on fertility and additive maternal effects on other traits failed to converge to reasonable values and were set to 0. Genetic trends were determined by regressing EBV means on year and testing the differences in the resulting regression coefficients between lines. Standard deviations of regression coefficients did not explicitly account for genetic drift, but effects of drift on EBV for fertility were approximated using procedures from [11].

## Results

### Fixed effects

**Fig. 3:**
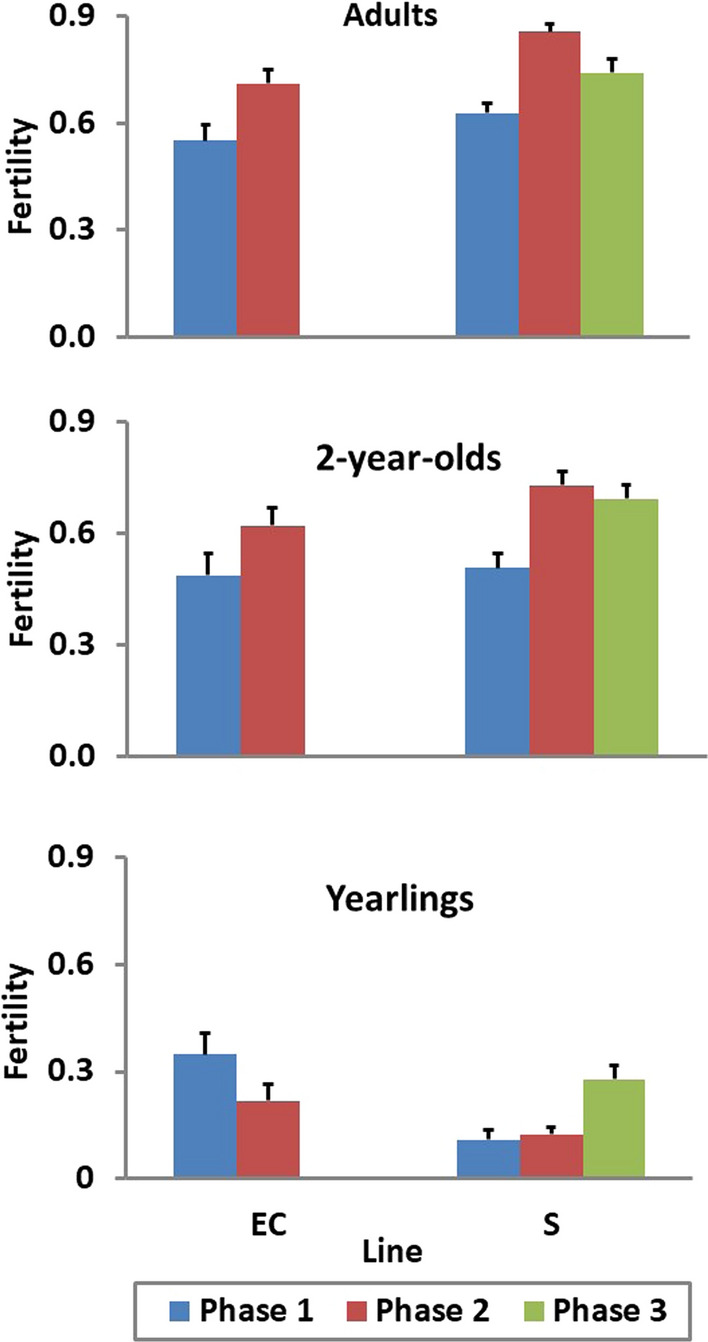
Phenotypic means for realized fertility in spring joining for yearling, 2-year-old, and adult (> 2-year-old) ewes in the selection (S) and environmental control (EC) lines in each phase of the study

Fertility differed with ewe age (Fig. 3). Adult ewes had higher fertility than 2-year-old ewes (P = 0.001), and both these groups had a much higher fertility than yearling ewes (P < 0.001). The average fertility of 14-month-old yearling EC ewes was higher than that of contemporary S yearling ewes (P < 0.001), which reflected their age difference at first joining. Average inbreeding coefficients for ewes mated in Phase 1 were on average 0.8% and did not differ among lines. In Phase 2, average inbreeding coefficients for mated ewes were on average 3.8% for the S, 3.0% for the EC, and 1.9% for the GC line. The smaller inbreeding coefficients found for the GC line reflected the lower ewe replacement rate in this line. The average inbreeding coefficient for S ewes that were mated in Phase 3 was 5.1%. Effects of inbreeding of the ewe on fertility and litter size and inbreeding of both the lamb and the ewe on BW and SC were negative, but small (P ≥ 0.20).

### Genetic parameters

The heritability of realized fertility during the spring joining periods was 0.07 ± 0.01 for the linear model and 0.15 ± 0.04 for the threshold model (Table 2). Permanent environmental effects of the ewe were also significant for fertility; repeatability estimates for fertility were 0.14 ± 0.02 for the linear model and 0.28 ± 0.03 for the threshold model. The random effect of service sire within mating year accounted for 7 ± 1% and 13 ± 2% of the phenotypic variation in fertility for the linear and threshold models, respectively. For litter size, the estimated heritability was 0.04 ± 0.02 and repeatability was 0.12 ± 0.03. Effects of the service sire accounted for only 1 ± 1% of phenotypic variance in litter size. Additive genetic, permanent environmental, and service-sire correlations between fertility (using the threshold model) and litter size were positive (0.24 ± 0.42, 0.49 ± 0.30, and 0.52 ± 0.48, respectively) but only the permanent environmental correlation was notably higher than its standard deviation (P = 0.10).

**Table 2:** Estimates and SD for phenotypic variances (${\sigma }_{\mathrm{P}}^{2}$) and variance components, as a proportion of ${\sigma }_{\mathrm{P}}^{2}$, obtained with single-trait models for fertility and litter size

Item	Fertility		Litter size
	Linear model	Threshold model	
Heritability	0.07 ± 0.01	0.15 ± 0.04	0.04 ± 0.02
Repeatability	0.14 ± 0.02	0.28 ± 0.03	0.12 ± 0.03
Service sire	0.07 ± 0.01	0.13 ± 0.02	0.01 ± 0.01
${\sigma }_{\mathrm{P}}^{2}$	0.189 ± 0.005	1.72 ± 0.11	0.34 ± 0.01

SD are the standard deviation of the parameter estimates from the posterior distribution. Litter size was fitted as a continuous variable using a linear model

The heritability estimate for lamb BW was low (0.05 ± 0.03) at birth but increased with lamb age to 0.19 ± 0.04 at 120 d (Table 3). Additive maternal, maternal permanent environmental, and litter effects were also significant for lamb BW. The heritability estimates for SC (Table 3) were larger at 90 d (0.41 ± 0.09) than at either 60 (0.31 ± 0.08) or 120 d (0.34 ± 0.09). Additive maternal and litter effects on SC were significant at all ages, but the additive maternal component may have been inflated by maternal permanent environmental effects that could be estimated separately.

**Table 3:** Estimates and SD for variance components, as a proportion of phenotypic variances (${\sigma }_{\mathrm{P}}^{2}$), for birth weight (kg) and for body weight traits (BW; kg) and scrotal circumferences (SC; cm) at 60, 90, and 120 days of age

Trait	Heritability	Maternal heritability	Maternal permanent environment	Litter effect	${\sigma }_{\mathrm{P}}^{2}$
Birth weight	0.05 ± 0.03	0.19 ± 0.03	0.20 ± 0.05	0.29 ± 0.03	0.7
60-day BW	0.11 ± 0.03	0.14 ± 0.03	0.04 ± 0.02	0.10 ± 0.03	15.5
90-day BW	0.15 ± 0.04	0.13 ± 0.03	0.05 ± 0.02	0.07 ± 0.03	25.6
120-day BW	0.19 ± 0.04	0.13 ± 0.03	0.06 ± 0.02	0.06 ± 0.03	33.3
60-day SC	0.31 ± 0.08	0.14 ± 0.04		0.12 ± 0.05	3.6
90-day SC	0.41 ± 0.09	0.17 ± 0.05		0.10 ± 0.05	10.6
120-day SC	0.34 ± 0.09	0.16 ± 0.04		0.09 ± 0.04	9.1

Estimates of heritability, maternal heritability, maternal permanent environmental effects, and litter effects were expressed as ratios of additive genetic (${\sigma }_{\mathrm{A}}^{2}$), additive maternal (${\sigma }_{\mathrm{M}}^{2}$), maternal permanent environmental (${\sigma }_{\mathrm{MPE}}^{2}$), and litter (${\sigma }_{\mathrm{L}}^{2}$) variances, respectively, to ${{\sigma }_{\mathrm{P}}^{2}=\sigma }_{\mathrm{A}}^{2}+{\sigma }_{\mathrm{M}}^{2}+{\sigma }_{\mathrm{MPE}}^{2}+{\sigma }_{\mathrm{L}}^{2}+{\sigma }_{\mathrm{e}}^{2}$ where ${\sigma }_{\mathrm{e}}^{2}$ is the residual variance. Parameters for birth weight were derived from a bivariate model that included fertility. Parameters for BW and SC were derived from four-trait multivariate models that also included fertility. Attempts to partition maternal effects on SC into additive maternal and maternal permanent environment components did not yield reasonable results, and the final model for SC contained only additive maternal effects. SD are the standard deviation of the parameter estimates from the posterior distribution

Both additive and maternal genetic correlations between BW at 60, 90, and 120 days all exceeded 0.89. Additive and maternal genetic correlations between SC at these ages averaged 0.90 and 0.77, respectively. Fertility in spring joining had genetic correlations of − 0.37 ± 0.30 with lamb birth weight, 0.09 to 0.15 with postnatal BW, and 0.02 to 0.10 with SC.

### Phenotypic trends

Least-squares means for fertility of yearling, 2-year-old, and adult S and EC ewes in each phase of the study (Fig. 3) were derived from a simple linear model that included fixed effects of line, year, ewe age (1, 2, or ≥ 3 years), and their interactions. Fertility of 2-year-old and adult ewes increased between Phase 1 and Phase 2 for both S and EC ewes but then, in Phase 3, did not change for 2-year-old S ewes and declined for adult S ewes. This apparent truncation of the selection response in the S line in Phase 3 was associated with the temporary exclusion of several ewes with high EBV from the breeding population for use in a series of intensive studies. However, in the final year of the study (2005), when all ewes were included in the breeding population, the mean fertility was 0.93 ± 0.07 for 2-year-old ewes and 0.88 ± 0.05 for adult ewes.

The difference between S and EC ewes in Phase 1 was 0.02 ± 0.07 (P = 0.38) for 2-year-old ewes and 0.08 ± 0.05 (P = 0.11) for adult ewes but increased in Phase 2 to 0.11 ± 0.06 (P = 0.08) for 2-year-old ewes and 0.14 ± 0.04 (P = 0.001) for adult ewes. Fertility of 2-year-old and adult S ewes in Phase 2 averaged 0.73 ± 0.09 and 0.86 ± 0.05, respectively. By comparison, fertility of spring-lambing yearling, 2-year-old, and adult GC ewes averaged 0.74 ± 0.04, 0.94 ± 0.02, and 0.93 ± 0.01, respectively. In yearlings, EC ewes had higher fertility than S ewes in Phase 1 (0.35 ± 0.06 versus 0.11 ± 0.03). However, the advantage in fertility for yearling EC ewes declined from 0.24 ± 0.07 (P = 0.001) in Phase 1 to 0.09 ± 0.05 (P = 0.08) in Phase 2, and fertility of yearling ewes in the S line further increased to 0.28 ± 0.04 in Phase 3.

### Genetic trends

**Fig. 4:**
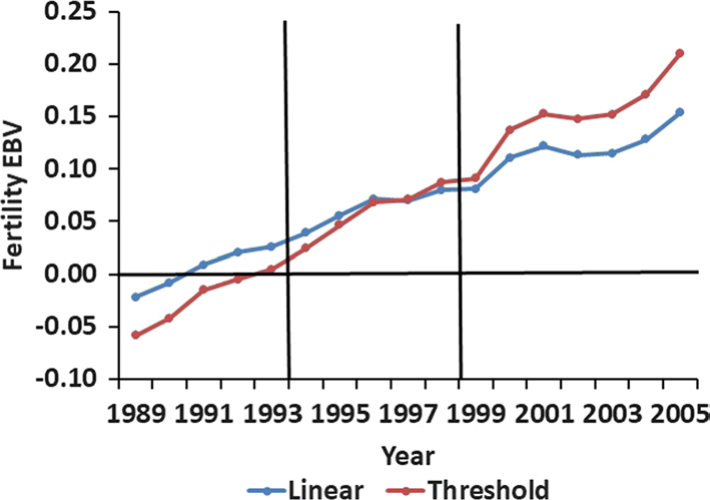
Additive genetic trends in fertility in the selection (S) line from a linear or a threshold model. EBV for fertility are averages for pairs of ewes and rams joined in each year. Vertical lines indicate the three phases of the study

Genetic trends in fertility in line S were based on the mean EBV for fertility of each pair of prospective parents (Fig. 4). As expected, estimates of heritability and selection response for fertility were higher with a threshold model than with a linear model. However, the correlation of EBV for fertility between the two models was 0.987, indicating that rankings of candidates for selection were not affected by the model used to derive EBV. For a linear model, EBV for fertility in line S improved from -0.022 ± 0.040 in 1989 to 0.153 ± 0.044 in 2005; the regression coefficient of EBV on year was 0.0101 ± 0.0004 per year. Rates of change in EBV for fertility were similar in Phases 1 and 2, but initially decreased in Phase 3, in association with the relaxed selection and use of S ewes in intensive studies. However, the mean EBV for fertility of S ewes increased in 2004 and 2005, when all available S ewes had opportunity to lamb.

**Fig. 5:**
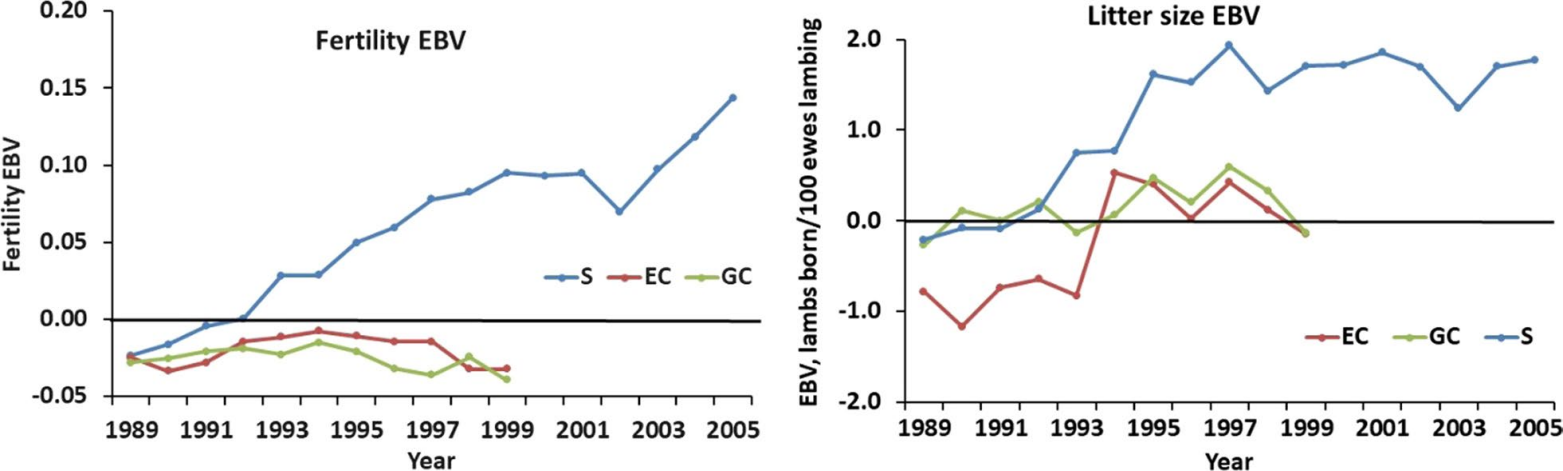
Additive genetic trends in fertility (ewes lambing in autumn per ewe joined in the spring) and litter size for each line. The EBV are averages for ewes joined for each line in each year

EBV for fertility of ewes in the S, EC, and GC lines were compared using EBV of ewes joined in each line and year (Fig. 5). The EC ewes were exposed to S rams in Phase 2, thus the use of parent-average EBV would have over-estimated the genetic trend in the EC line. Genetic trends in fertility were low for the GC (-0.0011 ± 0.0007 per year) and EC (0.0021 ± 0.0017 per year) lines. The genetic trend for the S ewes (Fig. 5) was slightly lower (0.0093 ± 0.0007 per year), particularly in Phase 3, than that based on parent-average EBV. The EBV for litter size increased by 0.0025 ± 0.0003 per year in line S in Phases 1 and 2, before plateauing in Phase 3 (Fig. 5). However, the total change in EBV for litter size in line S from 1989 to 2005 was only 0.020 lambs. The net change in EBV for litter size was trivial for both control lines.

**Fig. 6:**
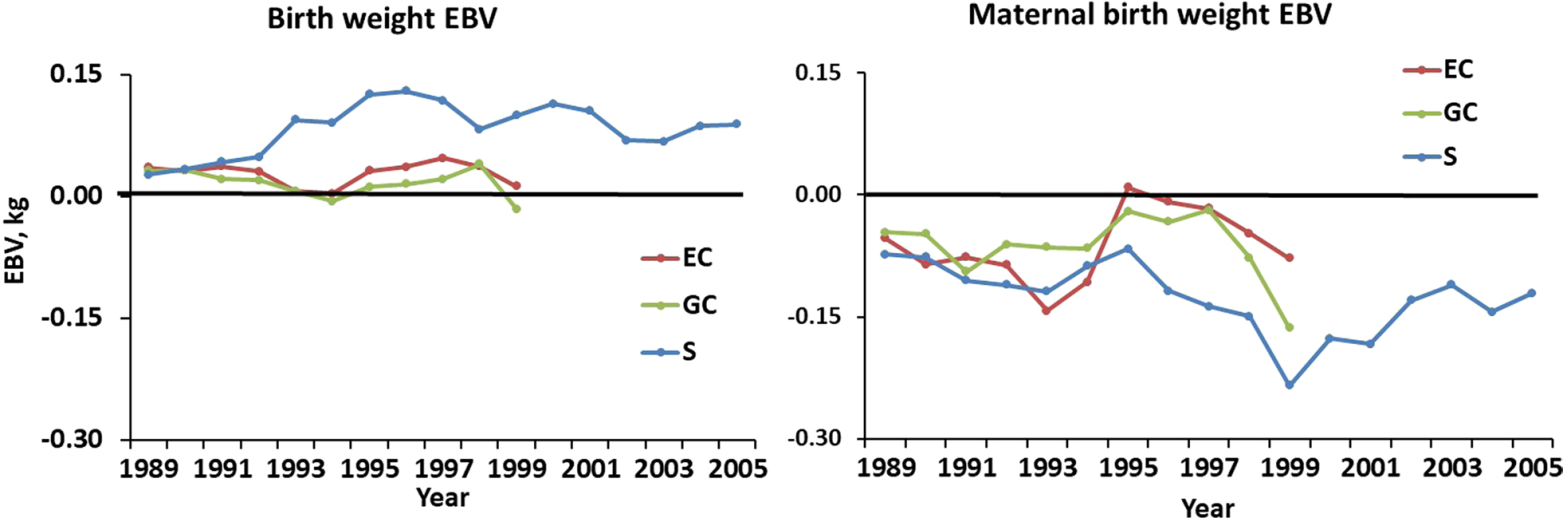
Direct and maternal additive genetic trends in birth weight. The EBV are averages for ewes joined for each line in each year

**Fig. 7:**
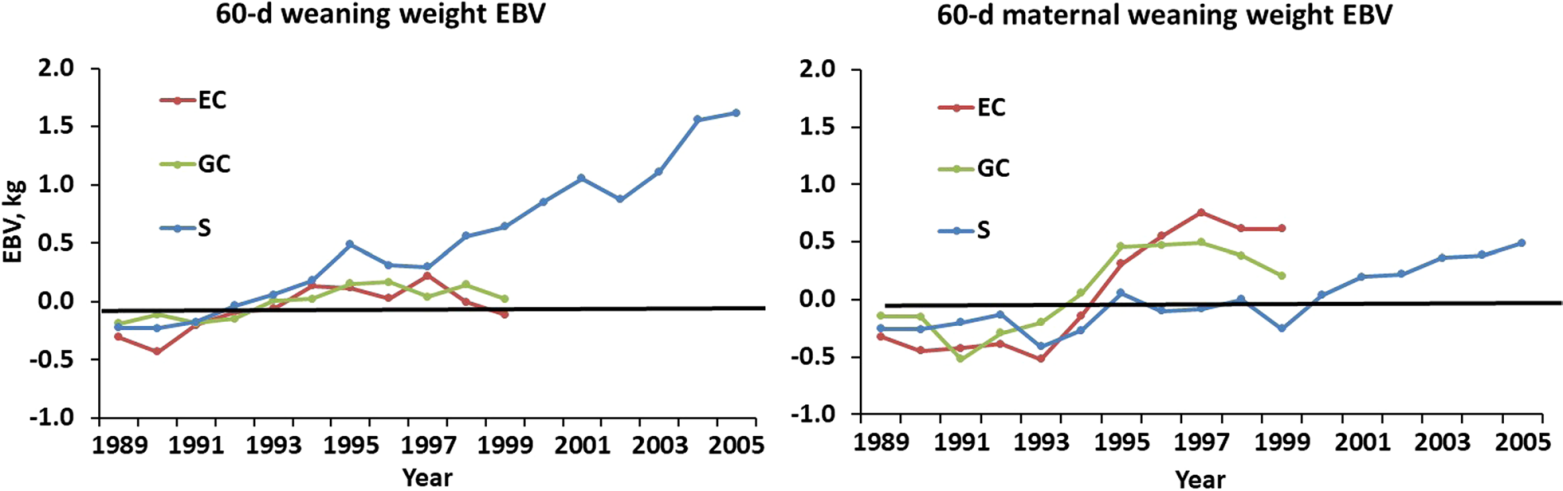
Direct and maternal additive genetic trends in lamb 60-day weaning weight. The EBV are averages for the ewes joined for each line in each year. Patterns of change in EBV for 90- and 120-d weights (not shown) were nearly identical to those for 60-d weight

**Fig. 8:**
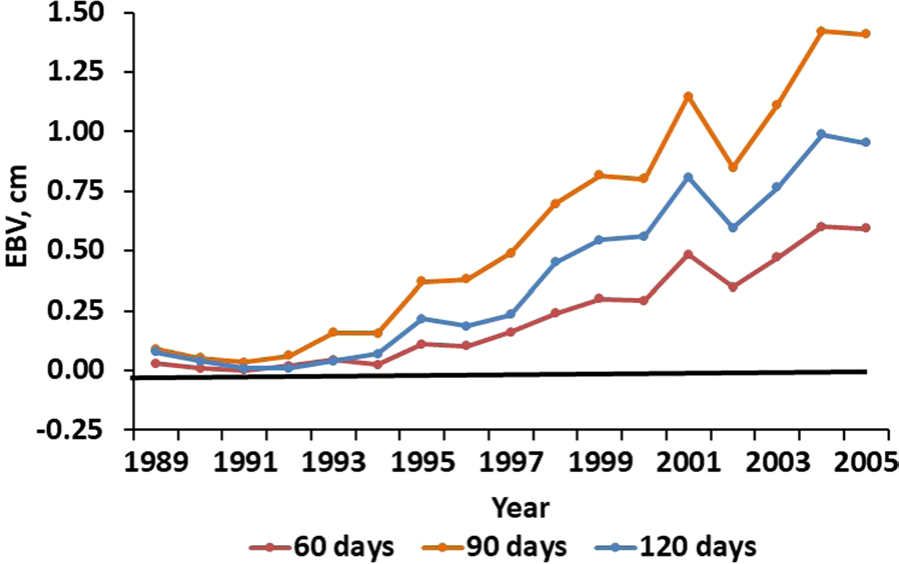
Additive genetic trends in lamb scrotal circumference (SC) in the selection (S) line at 60, 90, and 120 d of age. The EBV are averages for ewes joined in each year. At all measurement ages, additive genetic trends in SC in the environmental control (EC) and genetic control (GC) lines and additive maternal genetic trends in the three lines were negligible and are not shown

Additive genetic trends in EBV for birth weight were not observed in any of the lines (Fig. 6). However, a negative trend in EBV for maternal birth weight was observed for line S in Phases 1 and 2 (− 0.006 ± 0.002 kg/year) but reversed in Phase 3. Additive genetic trends in lamb BW were relatively small (Fig. 7). The linear trend in EBV for 60-day weaning BW in line S was 0.100 ± 0.006 kg (0.5%) per year but there was no detectable trend in additive maternal effects. Patterns of change in EBV for 90- and 120-day BW were essentially identical to those for 60-day BW, but EBV means and SE, and associated genetic trends were larger and proportional to additive direct and maternal standard deviations in Table 3. No changes in EBV for SC were observed in EC or GC lambs, and genetic trends in maternal EBV for SC were low for all lines and measurement ages (not shown). However, positive trends of 0.040 ± 0.003, 0.092 ± 0.006, and 0.065 ± 0.006 cm/year (0.3, 0.5, and 0.3%/year) were observed for SC in S lambs at 60, 90, and 120 days, respectively, with the highest genetic trend in SC observed at 90 days (Fig. 8).

## Discussion

This study documents the additive genetic effects on spring mating behavior in sheep. The heritability estimate for fertility during the spring joining period from the linear model was low (0.07 ± 0.01), but consistent with previous estimates of heritabilities of fertility in sheep [21]. However, the relatively low mean fertility and associated high phenotypic variation observed in our study resulted in useful levels of estimated additive genetic variation and a positive selection response. EBV for fertility in spring joining increased in selected ewes and, at the end of Phase 2, were higher for the S than the EC ewes. However, fertility of yearling ewes did not begin to improve until Phase 3 of the study. This pattern, and the apparent plateau in fertility of older ewes, was consistent with expectations for a threshold model, with the older ewes approaching the upper limit for mean fertility whereas improvements in breeding values for fertility on the underlying scale in yearling ewes allowed progressively more of them to become pregnant in the spring.

Over the 17 years of the study, fertility of line S increased by 0.01 per year (i.e., 2% of mean fertility) and the average EBV for fertility at the end of Phase 3 was 0.15 ± 0.03. Means of EBV for fertility from the linear model at the end of Phase 2 were 0.10 ± 0.01 for the S and − 0.032 ± 0.019 for the EC line. However, the SE for genetic trends and line differences in EBV for fertility did not explicitly account for genetic drift. Al-Shorepy and Notter [11] used procedures from [22, 23] to predict drift variances for fertility in S and EC lines at the end of Phase 1. These values were projected to the end of Phase 2 assuming proportionality of estimated drift variances to the duration of the study. Resulting predicted SE of the line means were 0.04 for line S and 0.03 for line EC, and the line difference at the end of Phase 2 was 0.13 ± 0.05 (P < 0.02). Thus, we concluded that improvement in additive genetic merit for fertility was achieved and that the sizes of the S and EC lines were, as intended, near the minimum required to meet the experimental objective.

A limitation of the experimental design was that EC ewes, born in March and joined for the first time in May at 14 months of age, were on average 7 months older than the S ewes at each joining. This problem could have been avoided by transferring frozen embryos from GC ewes into EC donors in the spring, but we rejected this option because of concerns regarding estrous induction and embryo transfer with frozen embryos in unselected ewes in spring. In retrospect, we see no better structure for the control lines.

The May joining corresponded to the anticipated maximum depth of anestrous at this location [2, 15]. However, intensive feeding of lactating ewes, creep feeding of suckling lambs, early weaning, and intensive feeding of weaned lambs helped to achieve a measurable selection response. Fertility of EC ewes in Phases 1 and 2 averaged 0.55 for adult ewes, 0.49 for 2-year-old ewes, and 0.35 for 14-month-old EC replacement ewes. These values compared favorably to published results for Finnsheep crossbred ewes joined in the spring [24, 25]. However, spring fertility of 8-month-old ewe lambs in line S averaged only 0.12 in Phases 1 and 2, but increased to 0.28 in Phase 3, which emphasizes the challenges involved in selecting replacement ewe lambs in production systems that involve autumn lambing [26]. Autumn-born lambs were anticipated to be genetically superior but were phenotypically unlikely to lamb at 1 year of age.

Several other studies attempted to reduce seasonality of reproduction in sheep [5–8], but few, if any, of these achieved unambiguous success. None included a contemporary unselected control, and most were terminated before a positive selection response was documented. However, differences among breeds of sheep in timing and duration of the breeding season are well-documented [1, 2], as well as breed differences in sensitivity of ewes to ram effect and the ability of rams to elicit this response [27, 28]. Thus, we were aware that selection could shift, rather than reduce, the seasonal anestrous or modify responses to the introduction of rams. However, several studies confirmed that S ewes had a longer breeding season than EC ewes. When ewes were exposed continually to vasectomized rams, negative (favorable) relationships were observed between EBV for fertility and duration of the seasonal anestrous [12, 14]. Ewes with high and low EBV were anestrous for an average of 28 and 70 d, respectively (P < 0.001), and some ewes with high EBV did not exhibit a seasonal anestrus [12]. The duration of the seasonal anestrus in selected ewes was not affected by continuous exposure to 16-h days in February through June [14]. In that study, the average duration of anestrus for adult ewes was 34 days, and ewes commonly missed only one or two estrous cycles in late July and August. When adult ewes were isolated from rams for one year, the mean period of anestrous (based on circulating progesterone levels) was shorter for S ewes (57 d) than for St. Croix (133 d) or Suffolk (140 d) ewes [15], but only one of 10 S ewes cycled continuously for one year. The longer periods of anestrous in [15] compared to those observed with continuous ram exposure suggested that isolation from rams extended the seasonal anestrous. However, for S ewes all three studies [12, 14, 15] reported anestrous periods that were among the shortest ever reported for temperate sheep breeds, demonstrating that the breeding season of S ewes was extended to include the early May joining period.

**Fig. 9:**
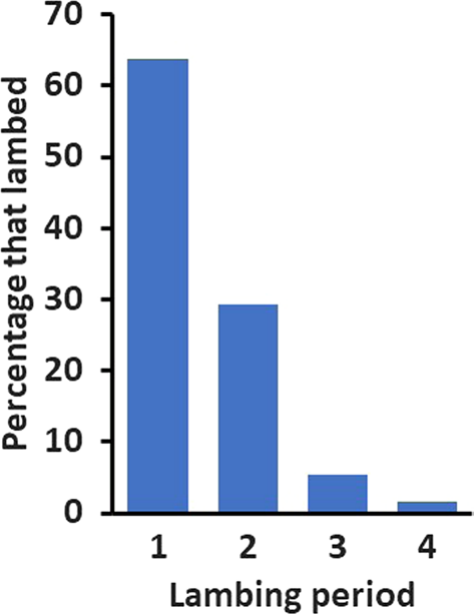
Distribution of lambing dates for selected ewes in Phases 2 and 3. Lambing periods represent consecutive 17-day periods (approximately corresponding to one estrous cycle) beginning 140 days after the start of the joining period

Changes in sensitivity to ram effect did not have a major impact on our results. Ewes were exposed to vasectomized rams in Phase 1 of the study, but not in Phases 2 or 3. For S ewes in Phases 2 and 3, most ewes lambed in the first 17 days of the lambing season (Fig. 9). By contrast, the first ovulation in ewes that were stimulated to ovulate by ram effect is usually not accompanied by estrus; i.e. the first estrus normally occurs at the second ovulation, resulting in a concentration of births at 17 to 23 days after the potential start of lambing [28]. The observed distribution of lambing dates indicated that most of the ewes that would eventually lamb were cycling at the time of ram introduction. The efficacy of ram effect increases as the ewes approach the end of anestrous [27], and we chose an end to the spring joining period that was expected to allow a reasonable ewe fertility even if ewes did not begin to cycle until June. The fact that, in our study, selection response was achieved by deferring the start of anestrous, rather than by relying on ram effect to accelerate the beginning of the next breeding season, was fortunate. Lambing was nearly complete by the end of October, and ewes that did not lamb early (i.e., that were not cycling at joining) were unlikely to lamb. Thus, weaning occurred in December and maximized the time available for ewes to recover from lactation before the next spring joining.

Mechanisms that control seasonal breeding in sheep are relatively well-understood [29] and involve translation of external changes in day length to circannual changes in neuro-endocrine responses. These changes are mediated by photoperiodic control of the timing and duration of melatonin secretion from the pineal gland by a variety of clock genes [30]. In this study, the control points that were disrupted to extend the breeding season are unknown. Ewes with high EBV for fertility had lower nocturnal melatonin levels and higher nocturnal prolactin levels in August than ewes with low EBV for fertility [13], but it is unlikely that these changes had a causal role in reducing the seasonal anestrous. A more likely explanation was that selection reduced stringency of the photoperiodic control over expression of the breeding season, allowing fluctuations in nocturnal melatonin levels. Spring fertility was associated with polymorphisms in the *melatonin receptor 1a* gene in S and EC ewes [31]. Similar associations were reported in other breeds, but a comprehensive review [32] suggested that these polymorphisms were at best breed-specific, rather than species-wide, indicators of genetic variation in seasonal breeding.

The objective of our study was to genetically improve ewe fertility during the annual spring joining period. However, there has also been interest in identifying methods to improve ewe reproductive efficiency in a wider range of out-of-season and accelerated lambing systems [33]. The potential performance of S ewes in accelerated lambing was evaluated by exposing lactating S ewes to rams in spring [16]. When lactating S ewes were joined with rams in March at approximately 60 d postpartum, 76% of the ewes became pregnant and 62% lambed, indicating only modest effects of season and lactation on pregnancy rates. However, when lactating S ewes were joined with rams at an average of 40 d postpartum in May, 53% of the ewes mated within 39 d of joining but only 21% of the ewes produced viable offspring, which suggest problems with maintenance of pregnancy and placental insufficiency. Selection to improve reproductive performance in accelerated lambing is challenging [26]. Our results suggest that genetic improvement of reproduction in accelerated and other multi-season lambing systems could be achieved as a correlated response to selection for fertility in May and June in nucleus flocks used to produce commercial replacement ewes.

Correlated responses to selection to improve fertility were modest. EBV for litter size increased by 0.1% per year through Phase 2 but plateaued in Phase 3. The heritability estimate of 0.04 ± 0.02 for litter size in the current study was lower than both the average heritability of 0.11 for litter size in sheep [21] and the estimate of 0.10 obtained by using data from Phase 1 of the current study [10]. These results suggest that expression of additive effects on litter size may have been constrained as selection for fertility progressed. Rapid rebreeding of lactating S ewes led to an enhanced frequency of fetal deaths, perhaps due to inadequate placental development [16]. Negative trends in EBV for maternal birth weights through Phase 2 could likewise be associated with greater fetal losses. Thus, we hypothesized that the association between ovulation rate and litter size may have been weakened in ewes that conceived in spring, thereby limiting expression of additive variation in litter size.

EBV for body weight at 60, 90, and 120 d increased by approximately 0.5%/year, perhaps in association with secondary selection for BW in replacement lambs. Screening ram lambs based on SC and mating behavior did not always favor the largest lambs, but it is likely that some positive selection on BW occurred. There were no significant correlated responses in maternal effects on postnatal BW.

Correlated responses in birth weight were more complex than those observed for postnatal BW. EBV for birth weight increased until 1996 but then slowly declined. However, maternal effects on birth weight declined in Phase 1 and, particularly, in Phase 2. Occasional problems with underweight lambs and reduced survival were observed in Phase 2. As a result, some positive emphasis was placed on EBV for maternal birth weight in the selection of ram lambs in Phase 3 and appeared to have a positive effect on lamb birth weight. These results were consistent with seasonal differences in birth weight in these populations and larger estimates of additive maternal variation for birth weight in autumn-born lambs [17]. Our findings, and those of Goff et al. [16], suggested that ewes that conceive in spring may be less able to maintain resulting pregnancies. Selection increased the likelihood that ewes would conceive and maintain pregnancy, but, initially at least, at a possible cost in terms of reduced lamb birth weights.

Correlated responses in SC were positive and largest (0.5%/year) at 90 d of age. Maximum testicular growth occurs at approximately 90 d of age [34], suggesting that selection to improve fertility accelerated sexual development. The impact of screening ram lambs for breeding soundness could not be quantified because of the small number of lambs that were evaluated, but it may have contributed to correlated responses in SC. Selection for serving capacity in ram lambs may also have contributed to changes in ewe fertility, but should not have biased comparisons between S and EC ewes in Phase 2, when all ewes were mated to the same rams.

## Conclusions

Our findings indicate that selection can be used to improve realized fertility (i.e., the proportion of ewes that subsequently lamb) in ewes joined in May and June and that changes in sensitivity to ram effect did not meaningfully contribute to the selection response. Genetic antagonisms with other performance traits were small, although a modest antagonism with maternal breeding values for birth weight may require attention in selection programs to maintain adequate lamb birth weights. The heritability of litter size in autumn lambing was lower than expected and could potentially limit the response to selection for prolificacy. This study is consistent with previous ones, i.e. that selection in these ewes shortened the seasonal anestrous by delaying the onset of anestrous until mid- to late summer with no corresponding delay in the onset of the next breeding season.

## Data Availability

The datasets used for the current study are available from the corresponding author on reasonable request.
